# Comment on “Fluorotechnology Is Critical to Modern Life: The FluoroCouncil Counterpoint to the Madrid Statement”

**DOI:** 10.1289/ehp.1510207

**Published:** 2015-07-01

**Authors:** Ian T. Cousins, Simona A. Balan, Martin Scheringer, Roland Weber, Zhanyun Wang, Arlene Blum, Miriam Diamond, Tony Fletcher, Gretta Goldenman, Christopher Higgins, Avery E. Lindeman, Graham Peaslee, Xenia Trier, Pim de Voogt

**Affiliations:** 1 Stockholm University, Stockholm, Sweden; 2 Green Science Policy Institute, Berkeley, California, USA; 3 ETH Zürich, Zürich, Switzerland; 4 Leuphana University, Lüneburg, Germany; 5 POPs Environmental Consulting, Schwäbisch Gmünd, Germany; 6 University of California at Berkeley, Berkeley, California, USA; 7 University of Toronto, Toronto, Ontario, Canada; 8 London School of Hygiene & Tropical Medicine, London, United Kingdom; 9 European Centre on Sustainable Policies for Human and Environmental Rights, Brussels, Belgium; 10 Colorado School of Mines, Golden, Colorado, USA; 11 Hope College, Holland, Michigan, USA; 12 Technical University of Denmark, Kongens Lyngby, Denmark; 13 University of Amsterdam, Amsterdam, the Netherlands

We commend the FluoroCouncil for phasing out long-chain poly- and perfluoroalkyl substance (PFAS) chemistry. However, members of the FluoroCouncil have been producing long-chain PFASs for decades while in possession of research showing adverse health effects in humans and animals. This model of chemical manufacturing needs to change. We recommend implementing the principles of green chemistry (Anastas and Warner 1998) in chemical manufacturing to ensure safer and sustainable chemical products. The scientific consensus of the Madrid Statement authors and signatories is that the use of all PFASs is unsustainable, and can and should be greatly reduced and discontinued where feasible. Short-chain fluorinated alternatives were therefore intentionally included in the scope of the Madrid Statement.

Some of the functionalities provided by fluorotechnology have become part of modern life. However, we disagree that PFASs are critical to modern life. Sustainable and less hazardous alternatives are available for many functionalities, and others will be developed. PFAS-based chemistries are used in many nonessential applications such as clothing, sports equipment, food packaging materials, blooming and dispersion agents, and stain-repellant treatments. We urge the FluoroCouncil to provide as much information as possible on the PFAS chemistries used in different commercial products and technologies.

We are aware that short-chain perfluoroalkyl acids bioaccumulate less than long-chain ones. However, some short-chain PFASs have been linked to adverse biological effects (Bull et al. 2014), and further systematic, representative studies on additional end points are needed. Given the ongoing release and environmental persistence of short-chain acids, increasing environmental and human exposures such as those documented by Glynn et al. (2012) are expected, for example, via contaminated drinking water aquifers (Xiao et al. 2015). Thus, continuous release of short-chain PFASs can be expected to lead to poorly reversible internal exposures, regardless of their low bioaccumulation potential (Scheringer et al. 2014).

Bowman commented that the Madrid Statement cannot claim insufficient data on the hazards and risks of fluorinated alternatives. However, Wang et al. (2015) highlighted the specific data gaps that prohibit conducting hazard and risk assessments for many fluorinated alternatives. An assessment commissioned by the FluoroCouncil (ENVIRON International Corporation 2014) also identified many gaps regarding human health data.

Bowman stated that “decisions on the societal acceptability of strategic materials such as PFASs cannot be wisely made on a single attribute such as persistence.” However, persistent chemicals are unsustainable in a world with limited resources. We cannot afford to “lose” portions of resources (water, soil, or food) because potentially harmful and persistent chemicals are accumulating over centuries and causing continuous exposure. Because of their persistence, an enormous inventory of PFASs is being created: Even if all PFAS production and uses were to stop immediately, PFASs would continue to be released for decades during products’ use and disposal life-cycle phases (Wang et al. 2014a, 2014b). One of the 12 principles of green chemistry is “design for degradation: chemical products should be designed so that at the end of their function they break down into innocuous degradation products and do not persist in the environment” (Anastas and Warner 1998). We endorse this principle and urge the FluoroCouncil to follow it also.

We welcome collaboration with the FluoroCouncil to establish information-sharing platforms for PFASs and support all opportunities for dialogue. We ask the FluoroCouncil to take leadership and responsibility for the global management of the PFASs they produce, from manufacturing to end of life.
